# Reprogramming cholesterol metabolism in macrophages and its role in host defense against cholesterol-dependent cytolysins

**DOI:** 10.1038/s41423-021-00827-0

**Published:** 2022-01-11

**Authors:** Min-Sub Lee, Steven J. Bensinger

**Affiliations:** 1 Department of Molecular and Medical Pharmacology, University of California, Los Angeles, CA 90095 USA; 2 Department of Microbiology, Immunology and Molecular Genetics, University of California, Los Angeles, CA 90095 USA

**Keywords:** Cholesterol, Macrophages, Innate Immunity, Metabolism, Innate immune cells, Mechanisms of disease

## Abstract

Cholesterol is a critical lipid for all mammalian cells, ensuring proper membrane integrity, fluidity, and biochemical function. Accumulating evidence indicates that macrophages rapidly and profoundly reprogram their cholesterol metabolism in response to activation signals to support host defense processes. However, our understanding of the molecular details underlying how and why cholesterol homeostasis is specifically reshaped during immune responses remains less well understood. This review discusses our current knowledge of cellular cholesterol homeostatic machinery and introduces emerging concepts regarding how plasma membrane cholesterol is partitioned into distinct pools. We then discuss how proinflammatory signals can markedly reshape the cholesterol metabolism of macrophages, with a focus on the differences between MyD88-dependent pattern recognition receptors and the interferon signaling pathway. We also discuss recent work investigating the capacity of these proinflammatory signals to selectively reshape plasma membrane cholesterol homeostasis. We examine how these changes in plasma membrane cholesterol metabolism influence sensitivity to a set of microbial pore-forming toxins known as cholesterol-dependent cytolysins that specifically target cholesterol for their effector functions. We also discuss whether lipid metabolic reprogramming can be leveraged for therapy to mitigate tissue damage mediated by cholesterol-dependent cytolysins in necrotizing fasciitis and other related infections. We expect that advancing our understanding of the crosstalk between metabolism and innate immunity will help explain how inflammation underlies metabolic diseases and highlight pathways that could be targeted to normalize metabolic homeostasis in disease states.

## Introduction

Macrophages are key players in the innate immune system and are tasked with responding to a diverse array of pathogens. Macrophages rely on recognizing pathogen-associated molecular patterns (PAMPs) through the engagement of pattern-recognition receptors (PRRs) and other similar receptors [1, 2]. Rapid recognition of foreign elements results in the production of proinflammatory cytokines and chemokines, phagocytosis, oxidative bursts to clear microbial pathogens, the activation of neighboring immune cells, and the recruitment of other immune cells to the site of infection [3]. Macrophages are also critical for the resolution of inflammation, the restoration of tissue homeostasis through their ability to remove cellular debris and apoptotic cells, and the coordination of wound repair processes [3]. The ability of macrophages to detect these distinct and disparate signals and subsequently integrate this information into physiological responses makes them ideal cells to mechanistically study how immune responses are regulated at the molecular level.

Macrophages rapidly reprogram their metabolic state to facilitate inflammation, resolution, and effector functions [4, 5]. The metabolic reprogramming of macrophages targets nearly all aspects of core metabolic pathways, including glycolysis, oxidative metabolism, the redox state, and nucleotide, protein, and lipid composition [4–6]. Without these coordinated changes in cellular metabolism, macrophages have deficits in their immune functions and contribute to disease pathogenesis in many contexts. This review focuses on one interesting and less understood aspect of metabolic reprogramming: the abrupt and profound shift in cellular cholesterol homeostasis induced by PRR signals and other proinflammatory stimuli. We provide a brief introduction to cellular cholesterol metabolism and the molecular mechanisms underlying the rapid alterations in cholesterol homeostasis as they are currently understood. We then discuss the potential reasons why inflammatory signals drive the intracellular redistribution of cholesterol in macrophages. We specifically highlight one interesting aspect of gram-positive bacterial infections that rely on membrane cholesterol to induce cytotoxicity and the resultant tissue damage. Finally, we discuss how manipulating cholesterol metabolism might be an attractive therapeutic approach to bolster host defense and spare tissues from pathogen-mediated damage.

## Regulation of cellular cholesterol homeostasis

Among the thousands of lipids found in the mammalian lipidome, cholesterol is the most abundant lipid species found in cells, accounting for up to 30% of total lipids [7]. Cholesterol is exceedingly hydrophobic and composed of four planar rings, and as such, it must reside within lipid bilayers of membranes or be stored in its esterified form within lipid droplets. Cholesterol is an essential lipid for all mammalian cells, playing indispensable roles in establishing cell membrane biochemical and biophysical properties, including the organization of lipid microdomains, receptor distribution, bilayer fluidity, and ultimately membrane integrity [7–9]. An interesting and important feature of cholesterol metabolism is that cholesterol can be synthesized within cells or directly imported by the internalization of lipoproteins [10]. Thus, requisite cholesterol levels can be met through the combined actions of synthesis and import pathways.

Cholesterol cannot be degraded by mammalian cells, and excess cholesterol is either stored in intracellular depots as lipid droplets or exported from the cell via the cholesterol efflux pathway [10]. Excess accumulation of cellular cholesterol can result in severe cellular dysfunction and activation of the inflammasome, leading to IL-1β-mediated inflammation [11]. Given the essential but potentially toxic nature of cholesterol, the biosynthetic, import, export, and esterification mechanisms must be tightly and coordinately regulated to ensure that sufficient but not excessive cholesterol is available to cells. Disrupted regulation of cholesterol metabolism is linked with many types of severe congenital human diseases, such as Tangier disease, familial hypercholesterolemia, Niemann-Pick type C disease, and Schnyder corneal disease [10]. Importantly, the balance of these pathways is rapidly altered in the context of inflammation, infections, autoimmunity, cancer, allergy, and wound repair [12].

### Cholesterol biosynthesis

The details of the cholesterol biosynthetic pathway are exceedingly complex but well defined [10]. Cholesterol is synthesized through the enzymatic activity of over 20+ distinct metabolic steps in the mevalonate pathway and the downstream cholesterol synthesis pathway. The enzymes involved in cholesterol biosynthesis are largely found in the endoplasmic reticulum (ER) membrane. These enzymes are subject to multiple layers of transcriptional and posttranslational regulation to ensure tight control over synthetic capacity and import to avoid deleterious accumulation [10].

The expression of cholesterol biosynthetic enzymes is transcriptionally regulated by the transcription factor, sterol regulatory element-binding protein 2 (SREBP2). SREBP2 has complex regulation, and we direct readers to excellent reviews that discuss the biology of this transcription factor [13, 14]. In brief, SREBP2 resides in the ER in association with two other proteins: SREBP-cleavage-activating protein (SCAP) and insulin-induced genes (INSIGs) [10, 15]. This protein complex is sensitive to ER sterol levels and controls SREBP2 function. Under sterol-replete conditions, SREBP2 is held in the ER, effectively decreasing cholesterol synthesis. When cholesterol levels in the ER drop below the cell threshold, SREBP2 translocates to the Golgi, where it is sequentially cleaved by site 1 and site 2 proteases (S1P and S2P, respectively) [10, 15]. Cleaved SREBP2 then moves to the nucleus to transactivate the genes encoding enzymes in the cholesterol synthesis program. In immune cells, the SREBP2 transcriptional program can also be activated by receptor-mediated kinase signaling pathways. For example, TCR, BCR, and select PRR signaling can increase cholesterol biosynthetic flux in both innate and adaptive immune cells, usually through the AKT/mTOR signaling pathway [16–19].

The expression levels of cholesterol biosynthetic enzymes are also directly regulated by the pool of intracellular sterols. The rate-limiting enzyme in cholesterol biosynthesis is the ER-residential protein, 3-hydroxy-3-methyl glutaryl coenzyme A reductase (HMGCR). Increased amounts of oxysterols in the ER, such as 25-hydroxycholesterol (25HC), can induce the proteolytic degradation of HMGCR, resulting in decreased cholesterol biosynthesis [10, 20]. In addition, cholesterol, 25HC, and other oxysterols potently retain the SCAP-SREBP2 complex in the ER, preventing SCAP-SREBP2 translocation to the Golgi body and the subsequent processing and nuclear import of SREBP2. Of particular importance in inflammation and macrophage biology, CH25H, the enzyme responsible for generating 25HC, is considered a canonical interferon (IFN)-regulated gene [21–23]. The IFN-mediated upregulation of CH25H and resulting production of 25HC appear to underlie many of the changes in cholesterol homeostasis observed in macrophages during inflammatory responses to viruses and some microbes [24–26]. The production of 25HC has also been shown to activate liver X receptors (LXRs) to promote the resolution of inflammation and effectively link the regulation of the SREBP2 and LXR pathways downstream of IFN receptor signaling [10, 21, 27, 28].

### Cholesterol import

Despite the intrinsic ability to synthesize cholesterol, immune cells may preferentially utilize cholesterol import via receptor-mediated endocytosis to meet their cholesterol requirements. The mechanistic details of cholesterol import have recently been reviewed elsewhere [10]. In brief, the import of cholesterol canonically occurs through the import of low-density lipoproteins (LDL) from the extracellular space via the low-density lipoprotein receptor (LDLR). LDLR is a ubiquitously expressed cell surface glycoprotein and is a direct transcriptional target of SREBP2; therefore, its function is directly correlated with SREBP2 activity [10]. Surface LDLR captures circulating LDL, which becomes incorporated into clathrin-coated vesicles that enter the endocytic pathway. Following endocytosis, LDL-carried cholesteryl esters are hydrolyzed by lysosomal acid lipases (LAL) in lysosomes, liberating free cholesterol. Cholesterol is then exported from the lysosomal lumen via the concerted actions of Niemann-Pick Type C1 (NPC1) and NPC2 [10, 29]. These free cholesterols are delivered to other subcellular compartments, such as the ER and plasma membrane (PM), for subsequent equilibration with the cellular cholesterol pool and distribution to other compartments of the cell as required [10].

### Cholesterol export

**Fig. 1:**
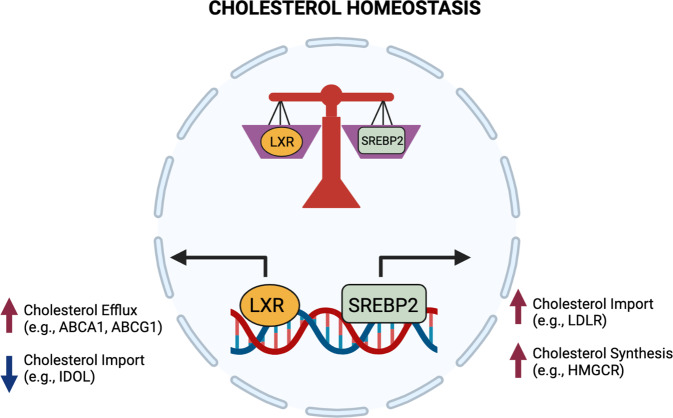
Opposing transcriptional programs ensure cholesterol homeostasis in macrophages. Cholesterol homeostasis is transcriptionally regulated by liver X receptors (LXRα and β) and sterol regulatory element binding transcription factor 2 (SREBP2) signaling. Excess cholesterol accumulation in cells activates LXRs, leading to increased cholesterol efflux and reduced cholesterol import. Activating the SREBP2 transcriptional pathway increases *de novo* cholesterol biosynthesis and increases cholesterol import

Unlike other lipid metabolites, such as long-chain fatty acids, phospholipids, and ceramides, mammalian cells do not have a built-in catabolic pathway for cholesterol. Instead, excess cholesterol in cells is either exported out of the cell or stored as cholesterol esters in lipid droplets. Cholesterol efflux is transcriptionally controlled by the lipid-activated nuclear receptors, liver X receptors (LXR α and β) [30]. Upon excess cholesterol accumulation in macrophages, LXRs transactivate the genes encoding the transporters *Abca1* and *Abcg1* [10]. The exact mechanism of ABCA1’s ability to move cholesterol out of the cell remains under investigation, but it is generally thought that ABCA1 flips phospholipids and cholesterol through the membrane, which then allows apolipoproteins (e.g., Apo-AI) to accept the newly accumulated cholesterol that now sits on the outer leaflet [31]. In contrast, ABCG1 is expressed intracellularly in endosomes, where it facilitates the movement of cholesterol from the ER to the inner leaflet via endosomal vesicles [32]. LXR activity, and correspondingly, ABCA1 and ABCG1 expression, can be altered by a wide array of inflammatory signals in macrophages. LXRs and SREBP2 help establish macrophage cholesterol homeostasis (Fig. 1). Importantly, we now know that proinflammatory signals control both the LXR and SREBP transcriptional axes, supporting the idea that rapidly modulating cholesterol synthesis and efflux in innate immune cells facilitates their effector function [17, 21, 27].

### Cholesterol esterification

As mentioned previously, cholesterol esterification is an important method for storing excess cholesterol in lipid droplets. Two major isozymes regulate cholesterol esterification: acyl coenzyme A:cholesterol acyltransferase 1 (ACAT1) and ACAT2. ACAT1 is highly expressed throughout the body, whereas ACAT2 has a more restricted expression pattern [10]. ACAT enzymes utilize free cholesterol as an allosteric activator and a substrate, subsequently conjugating fatty acyl-CoA into the hydrophobic end of the cholesterol molecule [10, 33]. This conjugation results in the production of cholesteryl esters, which become stored inside lipid droplets. Interestingly, neither ACAT1 nor ACAT2 contains regulatory elements specific for the transcription factors SREBP2 and LXR. However, the *Soat1* and *Soat2* genes that encode the cholesterol esterification enzymes ACAT 1 and 2, respectively, are upregulated by cytokines such as interferons and tumor necrosis factor, indicating a role for esterification in controlling inflammation and host defense [21, 34, 35].

## New concepts in plasma membrane cholesterol homeostasis

Cholesterol is essential for plasma membrane function. In the absence of sufficient plasma membrane cholesterol, cells cannot maintain lipid bilayer integrity and have perturbed microdomain assembly, thereby influencing signaling and endocytosis, among other functions. Although cholesterol in the plasma membrane represents the largest cholesterol pool within the cell, cholesterol is unevenly distributed throughout the plasma membrane. Accumulating evidence indicates that several distinct pools of cholesterol can be found within the plasma membrane, each serving unique roles in PM function and cellular physiology [36]. Recent elegant biochemical studies that leveraged microbial products that selectively bind to cholesterol in membranes have shed light on this exciting and important concept [37–39].

**Fig. 2:**
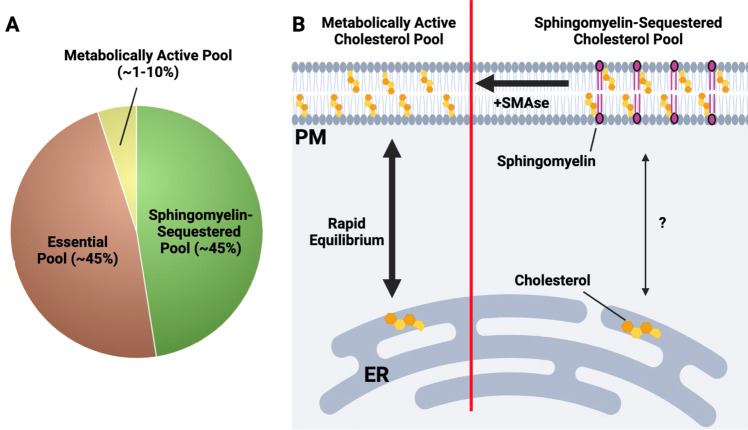
An overview of the three-cholesterol pool model in the plasma membrane. **A** The plasma membrane (PM) contains at least three distinct pools of cholesterol. The metabolically active or accessible cholesterol pool makes up ~1–10% of total PM cholesterol. Sphingomyelin-sequestered cholesterol and the essential pool are each estimated to be ~45% of total PM cholesterol. **B** Depiction of the metabolically active and sphingomyelin-sequestered cholesterol pools. On the left side of the diagram, the metabolically active cholesterol pool rapidly equilibrates with the ER cholesterol pool, thereby providing a metabolic conduit for ER-resident cholesterol sensing machinery (e.g., SCAP-SREBP, HMGCR) to sense PM cholesterol levels. On the right, the sphingomyelin-sequestered cholesterol pool is composed of cholesterol molecules that are tightly associated with sphingomyelin and likely other phospholipid species. In contrast with the metabolically active cholesterol pool, the SM-associated cholesterol pool does not rapidly equilibrate with the ER cholesterol pool. Treatment of cells with bacterial sphingomyelinase (SMase) liberates SM-associated cholesterol, allowing it to enter the metabolically active pool. The essential pool (not shown) is less well understood and thought to play a fundamental role in the bilayer integrity of the plasma membrane

Based on these studies, it is thought that the plasma membrane contains at least three distinct pools of cholesterol [36] (Fig. 2). One pool is termed the accessible or metabolically active pool. This fraction of PM cholesterol is very small and appears to be highly labile, meaning that the pool size can be rapidly altered in response to changes in total cellular cholesterol concentrations. This metabolically active pool is thought to be in equilibrium with the cholesterol pool in the ER and thereby influences the cholesterol sensing apparatus in the ER (e.g., SCAP/INSIG-SREBP). As such, the accessible cholesterol pool in the PM plays a critical role in linking PM cholesterol levels with the cholesterol pool in the ER and helps to set overall cellular cholesterol homeostasis. The exact distribution of the metabolically active pool of cholesterol in the membrane remains unknown, and further work is needed to determine if this pool is spatially restricted across the PM.

The importance of the metabolically active pool of cholesterol in pathogen lifecycle and host defense processes requires further investigation. Nevertheless, we suspect that this pool will be a critical target for microbes and viruses that exploit cholesterol metabolism as part of their pathogenesis. Indeed, it is worth noting that mechanistic work on this distinct cholesterol pool in the PM of cells relies on specific domains of microbial proteins that directly bind cholesterol (e.g., domain 4- of cholesterol-dependent cytolysins) [40]. Thus, it seems reasonable to infer that controlling specific cholesterol pools in the PM with inflammatory cytokines or other host defense machinery will be a component of innate immune responses to microbes and viruses. This concept is further explored below.

A second, larger pool of cholesterol in the PM is tightly associated with sphingomyelin (SM) and other phospholipid (PL) species [36]. This pool is known as the SM-sequestered pool, and the tight association of cholesterol with sphingomyelin forms the biophysical basis for the assembly of lipid microdomains or lipid rafts within the plasma membrane [41]. Similar to work using CDCs to define the metabolically active cholesterol pool, this distinct pool of cholesterol can be identified using the fungal-derived protein ostreolysin A (OlyA) [42]. The SM-sequestered pool size does not rapidly change in response to acute cellular cholesterol deprivation or overload. Thus, it is thought that the SM-sequestered pool does not directly equilibrate with the ER cholesterol pool or the metabolically active pool. However, brief treatment of cells with bacterial sphingomyelinase (SMase), which hydrolyzes sphingomyelin into ceramides and phosphorylcholine [43], decreases OlyA binding and rapidly increases the size of the accessible cholesterol pool. This finding indicates that newly liberated cholesterol from the sphingomyelin-associated pool flows into other PM-resident pools [36, 43]. This observation may be particularly relevant for microbial infections and the potential interplay between SMases and other toxins that rely on cholesterol for their effector functions.

The remaining cholesterol appears to exist in a third pool known as the essential pool. This cholesterol pool appears to be equivalent in size to the SM-sequestered pool, but it has been largely defined through exclusionary characteristics [36]. The exact nature of the essential pool of cholesterol remains poorly understood. However, we can hypothesize that this pool plays a fundamental role in bilayer integrity through its ability to foster molecular interactions between different classes of phospholipid species (i.e., PC, PE, PI, and PS) and other bilayer-associated proteins to establish fundamental biophysical characteristics [36]. The essential cholesterol pool requires harsh chemical treatment of cells to remove it from the membrane, and its removal results in the loss of cellular viability. The regulation of the essential pool size and the spatial distribution of this pool within the membrane have not been clearly defined. There is the idea that microbes and viruses specifically target distinct pools of cholesterol for their pathogenesis. However, there is no direct evidence that the essential pool is specifically targeted or exploited by pathogens to facilitate their lifecycle.

## Metabolic reprogramming of cholesterol homeostasis in macrophages

**Fig. 3:**
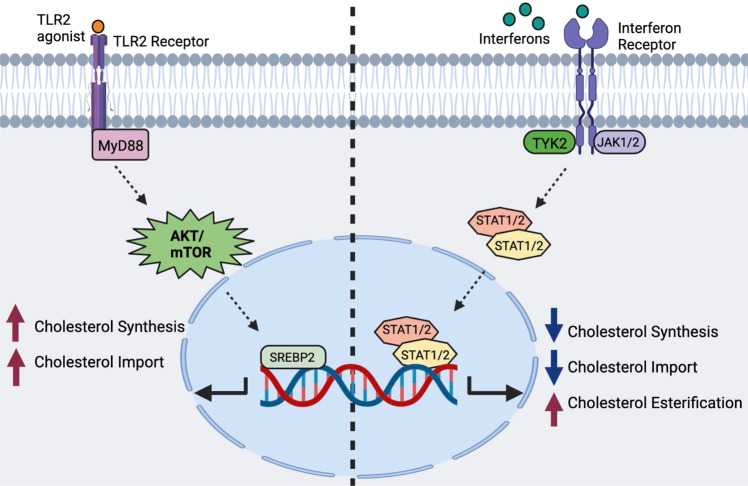
Signal-specific reprogramming of cholesterol metabolism in macrophages by proinflammatory signals. On the left side, macrophages receive an input stimulus that signals through MyD88-dependent PRRs (e.g., TLR2). MyD88 signaling activates AKT/mTOR, which increases SREBP2 transcriptional activity, resulting in increased cholesterol biosynthesis and cholesterol import. On the right side: type I and II IFN signaling reprograms macrophage cholesterol metabolism in a JAK-STAT-dependent manner. IFN receptors drive the downregulation of cholesterol biosynthesis, reduce cholesterol import, and upregulate cholesterol esterification

It is well understood that many types of immune cells reprogram their cholesterol metabolism in response to activation and proinflammatory signals. The activation of lymphocytes results in the coordinated upregulation of both cholesterol biosynthesis and lipoprotein import to meet the anabolic lipid requirements associated with growth and proliferation. In other contexts, phagocytes reprogram their cholesterol metabolism in a signal-specific manner, resulting in distinct metabolic outcomes [17]. Lipidomics and isotope tracer analyses have revealed that macrophages rapidly upregulate cholesterol synthesis when activated through MyD88-dependent PRRs, resulting in the general accumulation of total cellular cholesterol [17, 21]. In contrast, interferon (IFN) signaling and PRRs that generate IFN responses (e.g., TLR3-TRIF signaling) downregulate cholesterol biosynthesis and increase cholesterol storage in the form of cholesterol esters [21, 44]. Thus, it has been proposed that macrophages shift their cholesterol metabolism in a context-specific manner to facilitate specific effector functions and ensure the generation of particular forms of inflammation (i.e., IFNs versus IL-1β) (Fig. 3).

Interestingly, changes in synthesis and total cellular cholesterol levels do not necessarily correlate. For example, interferon signaling (both type I and II) decreases cholesterol biosynthesis via transcriptional and posttranslational mechanisms. Despite a decrease in cholesterol biosynthetic capacity, macrophages maintain or modestly increase their cholesterol levels in response to IFNs [21, 45]. This observation suggests that interferon signaling regulates other cholesterol homeostatic pathways (e.g., increasing cholesterol import or reducing cholesterol efflux pathways). Further analysis of plasma membrane cholesterol pools suggests that interferon signaling specifically decreases the metabolically active pool of cholesterol in the plasma membrane without disturbing the sphingomyelin-sequestered cholesterol pool or the essential pool [21]. The molecular events underlying selective reprogramming of plasma membrane cholesterol pools remain a poorly understood but is an active area of investigation. It is known that some aspects of IFN-mediated reprogramming are dependent on the production of oxysterol 25HC [10]. The capacity of 25HC to specifically decrease the accessible cholesterol pool is thought to increase cholesterol esterification, decrease cholesterol biosynthesis, and activate the LXR pathway [10, 34, 46]. This observation further highlights the exquisite specificity by which IFNs can remap the cellular cholesterol landscape and indicates that shifting specific cholesterol pools of the cell is an important component of host defense against viruses and aspects of microbial infections.

In contrast to IFN signaling, MyD88-dependent PRRs, such as Toll-like receptor (TLR)-2, TLR-7, and TLR-9, lead to an overall increase in cholesterol biosynthesis and total cholesterol in macrophages [21]. This increase in cholesterol is dependent on the upregulation of the SREBP transcriptional axis. The exact mechanism linking MyD88 signaling with the SREBP transcriptional axis has yet to be fully elucidated but is dependent on AKT/mTOR and likely the transcription factor, NRF2 [17]. Due to increased cholesterol synthesis, MyD88-dependent PRRs expand the accessible cholesterol pool in the plasma membrane [21]. Despite this increase in total and membrane cholesterol levels, MyD88-dependent PRRs do not significantly increase the levels of cholesterol esters. Thus, this reciprocal relationship between MyD88 and IFN signaling in cholesterol homeostasis in macrophages supports the concept that rapid and profound regulation of cholesterol homeostasis is an integral aspect of macrophage’s ability to respond to a broad array of pathogens and inflammatory stimuli.

## Cholesterol and bacterial pathogenesis

The conserved use of cholesterol in mammalian cells likely underlies cholesterol targeting by microbes and viruses to facilitate their lifecycle and pathogenicity. These strategies have been described in several reviews, and we direct the reader to the literature to gain a greater appreciation of the specifics of the different classes or types of pathogens [47, 48]. In this brief review, we restrict our discussion to some interesting aspects of cholesterol metabolism in bacterial pathogenesis, followed by a more detailed discussion of cholesterol-dependent cytolysins (CDCs), a family of pore-forming toxins that rely on cholesterol for their effector functions.

One well-characterized example of the interplay between cholesterol metabolism and microbial pathogenesis can be found in *Mycobacteria* infections. One hallmark of *M. tuberculosis (M. tb)* infection of cells is the marked accumulation of intracellular lipids resulting in foam cell formation [49]. It has been shown that *M. tb* has the ability to degrade cholesterol for use in bacterial metabolism and that this cholesterol catabolism may be a mechanism by which *M. tb* persists in IFN-activated macrophages [50]. Similarly, *M. leprae* uses the oxidation of cholesterol to facilitate energetics and cell wall biosynthesis [51]. Importantly, interfering with the ability of these bacteria to use cholesterol decreases intracellular survival in host cells. Thus, cholesterol is a requisite host metabolite that is used as a nutrient source by *Mycobacteria* for persistence and pathogenicity.

In other instances, obligate intracellular bacteria use cholesterol to facilitate entry into host cells. The hydrophobic nature of cholesterol means that this lipid is primarily embedded in cellular membranes. Some intracellular microbes specifically target the pool of cholesterol in cholesterol-rich microdomains in the PM for their entry [48]. Microbe-induced alterations in cholesterol metabolism have also been shown to perturb cellular phagolysosome function and intracellular organelle trafficking [48, 52]. Pharmacologic or genetic disruptions of lipid microdomains or intracellular cholesterol trafficking pathways decreases the efficiency of microbe entry and intracellular pathogen persistence [48, 53]. While these are just a few examples of how microbes exploit host cholesterol metabolism for their pathogenesis, they support the concept that reprogramming cholesterol homeostasis through inflammation is an innate immune mechanism used in host defense. These observations also support the idea that pharmacologically targeting cellular cholesterol metabolism could be an adjunctive therapeutic approach to facilitate the clearance of microbes.

## Cholesterol-dependent cytolysins

As discussed previously, microbial proteins can target lipids in membrane to facilitate pathogenesis. One well-defined group of virulence factors that target host cholesterol are cholesterol-dependent cytolysins (CDCs). CDCs are pore-forming toxins that are secreted as soluble monomers and subsequently oligomerize on host membranes to form pores [40]. Approximately thirty distinct gram-positive bacteria have been identified that produce CDCs, including several species that mediate severe diseases in humans (e.g., *Clostridium perfringens, Streptococcus pyogenes, and Bacillus anthracis*) [54, 55]. CDCs contain four domains. Domains 1–3 primarily play structural roles that facilitate oligomerization into a prepore intermediate once cholesterol recognition occurs. Domain 4 contains a tryptophan-rich region(s) that is involved in cholesterol recognition and membrane binding. Once Domain 4 successfully binds to cholesterol in the membrane, the CDC monomers oligomerize into a prepore intermediate, which then becomes inserted into the membrane [56].

The cellular consequences of CDCs vary depending on the CDC dosage, duration, and cell type. CDCs create a pore that is ~250 Å in diameter, which is large enough to allow the leakage of biomolecules (amino acids, nucleotides, and small proteins), as well as ions (Ca^2+^, K^+^, Na^+^, and Cl^−^) [56]. Pores also allow the influx of water into cells, which leads to blebbing and apoptosis due to osmotic shock. The mechanisms underlying CDC-mediated cell lysis have been characterized in erythrocytes because these cells have a minimal capacity to repair membrane damage. Unlike erythrocytes, nucleated cells have an intrinsic membrane repair system that is triggered upon Ca^2+^ influx during pore formation [56]. Repair processes induced by CDC pore formation include patch repair, clogging, and microvesicle shedding [56, 57]. Whether changes in cholesterol metabolism induced by microbes or host inflammation alter the efficiency of membrane repair is largely unknown and should be investigated. However, it is easy to hypothesize that the dramatic alterations in lipid homeostasis observed in response to proinflammatory signals will have some impact on host membrane repair systems.

## Induced resistance to CDC toxicity via metabolic reprogramming

The specific dependency of CDCs on membrane cholesterol to execute their effector function led to the hypothesis that alterations in membrane cholesterol homeostasis could provide some form of resistance to CDC-mediated cellular damage. Indeed, induced cholesterol efflux through pharmacologic activation of the LXR transcriptional pathway or genetic manipulation of cholesterol metabolism provides some measure of protection against CDC-mediated cellular toxicity [21, 46]. However, it is unclear whether physiological signals in the context of inflammatory responses to infections would have similar protective effects. Leveraging previous work that showed that PRR and cytokine signaling influence macrophage cholesterol homeostasis, we explored whether activating macrophages might intrinsically induce resistance to CDCs. Working from the hypothesis that the recognition of gram-positive bacteria by PRRs would be required for such a mechanism, we set out to determine whether activating macrophages with TLR2 agonists could influence CDC-mediated loss of membrane integrity. However, we found that this supposition was incorrect and that the opposite appeared to be true. We observed that activating macrophages via TLR2 and other MyD88-dependent TLRs resulted in a modest but highly reproducible increase in sensitivity to CDC-mediated cellular toxicity. Thus, TLR2-mediated recognition of gram-positive bacteria by macrophages does not appear to induce resistance to CDCs. Rather, this signal primes heightened CDC sensitivity. The increased sensitivity to CDCs is dependent on the ability of TLR2 to drive cholesterol biosynthesis, and inhibiting this pathway ameliorates the increase in CDC toxicity. Whether this circuit of TLR2 agonism, heightened cholesterol synthesis and increased sensitivity to CDCs is important for microbial pathogenesis remains to be tested [21].

In our studies on PRRs, we were surprised to find that TLR3 activation rendered macrophages very resistant to CDC-mediated cytotoxicity. These data led us to surmise that type I IFNs mediated this resistance and that TLR3 was an “accidental” mechanism by which we discovered this interesting response. Indeed, subsequent gain- and loss-of-function studies showed a critical and essential role for type I IFN signaling in this protective mechanism [21]. The activation of PRRs that lead to type I interferon production [58] (e.g., NOD2 and STING) also induced protection against CDCs. Importantly, delivery of either type I IFN or type II (IFN-γ) in trans resulted in protection, expanding the impact of this immunometabolic reprogramming event. We observed that IFN-stimulated macrophages retained functionality, even when challenged with CDCs, as evidenced by their ability to phagocytose microbes or apoptotic cells. We also found that the protective effect of IFNs could be observed in freshly isolated neutrophils, indicating that a generalized cellular mechanism, at least for phagocytes, underlie this response [21]. Whether this effect is also true for nonimmune cells stimulated with IFNs has not been determined.

The molecular mechanism of IFN-mediated protection against CDCs also lies in the ability of IFNs to alter cholesterol synthesis in macrophages. Isotope labeling studies showed that IFN signaling decreases cholesterol synthesis and that this decrease in cholesterol synthesis was dependent on the upregulation of CH25H and the subsequent production of 25HC [21]. The generation of 25HC by macrophages results in inhibition of the SREBP2 transcriptional axis and the direct degradation of HMGCR [20, 59, 60]. Consistent with this finding, genetic ablation of CH25H rendered naïve macrophages highly sensitive to CDCs and abrogated the ability of IFNs to protect against CDC-mediated pore formation. *Ch25h*-deficient mice also developed severe erythema and larger ulcerative skin lesions when intradermally challenged with streptolysin O (SLO), a CDC secreted by *S. pyogenes*. Conversely, pharmacologic addition of 25HC provided a marked level of protection against CDC challenge, solidifying the role of CH25H in this interesting immune-metabolic response. Consistent with a role for oxysterols in mediating protection to CDCs, both 25HC and 27HC protect endometrial cells from the CDC pyolysin, which is produced by *Trueperella pyogenes* [46]. Interestingly, this effect was partially dependent on the ability of these oxysterols to activate LXRs and reduce accessible cholesterol, likely through cholesterol efflux.

The molecular events mediating the ability of IFNs to protect against CDCs remain incompletely defined, but we have been able to gain some understanding of the pathways required for this effect. Using fluorescently labeled ALO-D4 (the D4 domain of the CDC Anthrolysin O) [39], we were able to show that IFNs decreased ALO-D4 binding to the membrane. These data suggest that the cholesterol levels in the plasma membrane dropped below those required for effective CDC binding and oligomerization [21]. Reprogramming cholesterol synthesis appears to be important for altering CDC binding to the plasma membrane, but how these changes in cholesterol synthesis directly translate into protection at the PM remains less clear.

One possibility is that inhibiting cholesterol biosynthesis globally reduces cholesterol levels in the PMs of cells. However, mass spectrometry data showed that cholesterol levels in the PM were largely maintained in IFN-stimulated macrophages, and we observed decreases in ALO-D4 binding as little as two hours after IFN treatment. Likewise, a brief 20-minute treatment of macrophages with sphingomyelinase, which effectively liberated cholesterol associated with sphingomyelin, quickly restored CDC binding and sensitivity to CDC-mediated toxicity. Thus, we concluded that a small and difficult-to-quantify cholesterol pool in the PM must be rapidly decreased in response to IFN signaling to mediate protection [21]. These data also suggest that the production of, microbial sphingomyelinases [61] in the context of polymicrobial infections will sensitize host cells to the harmful effects of CDCs and quickly overcome the protective effects induced by IFNs. It has also been shown that IFN signaling, downstream of TLR4, results in the accumulation of lanosterol, a sterol intermediate of the cholesterol biosynthetic pathway, in the PMs of macrophages [62]. This increase in lanosterol levels alters membrane fluidity, which potentiates phagocytosis by macrophages and the killing of *E. coli* [62]. Therefore, it remains possible that the accumulation of lanosterol or other sterol intermediates in the PM in response to IFN signaling contributes to this protective effect, perhaps through the dilution of the accessible cholesterol pool. However, this hypothesis needs to be formally tested.

It also remains unclear where the cholesterol targeted by CDCs is moved in response to IFN signaling. One possibility  is that cholesterol moves into another cholesterol pool within the plasma membrane. We measured sphingomyelin-associated cholesterol by imaging macrophages with the mushroom toxin protein, ostreolysin (OlyA), to test this possibility. In contrast to ALO-D4 staining, imaging macrophages with recombinant OlyA protein revealed little difference in staining between the IFN and control groups [21]. Thus, it does not appear that cholesterol from the CDC-targeted pool flows into the sphingomyelin-associated pool in response to IFNs. We were unable to test whether cholesterol moves into the essential pool since we cannot define this pool in macrophages. Additional biochemical studies on membrane fractions will be required to determine whether the lateral movement of cholesterol occurs in response to IFN and mediates protection of the PM to CDCs.

**Fig. 4:**
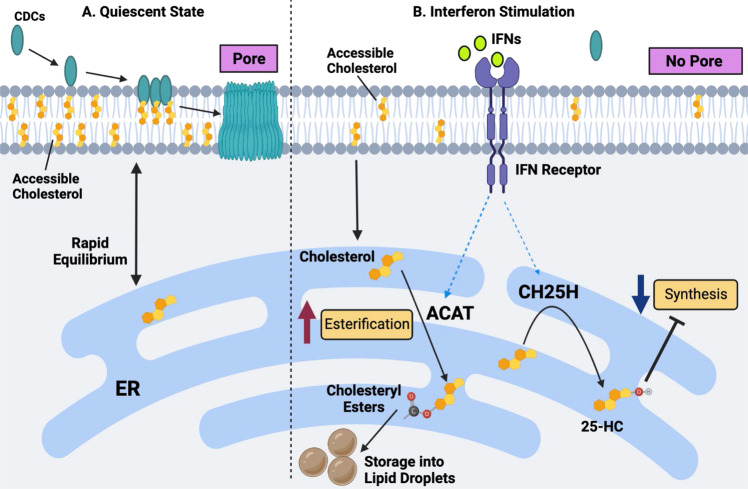
A working model of interferon-mediated protection of macrophages against CDC-induced cytotoxicity. **A** In a quiescent state, CDCs target metabolically active (or accessible) cholesterol in the plasma membrane of macrophages, resulting in pore formation and the subsequent loss of membrane integrity. **B** IFN stimulation markedly decreases the size of the accessible cholesterol pool, resulting in reduced CDC binding and pore formation on the plasma membrane. Alterations in the accessible cholesterol pool in the plasma membrane are driven by a reduction in cholesterol biosynthesis and heightened cholesterol esterification. The inhibition of cholesterol synthesis and esterification is dependent, in part, on the upregulation of the interferon-stimulated gene, *Ch25h*, and the production of oxysterol 25HC. 25HC decreases synthesis via the degradation of the HMGCR enzyme and the inhibition of the SREBP2 transcriptional pathway. 25HC also facilitates cholesterol esterification via the ER-residential enzymes ACAT1 and ACAT2.

An alternative explanation is that the cholesterol required for CDC recognition is rapidly moved into another subcellular location. In support of this concept, we observed that IFNs upregulated several genes involved in intracellular cholesterol movement (e.g., *Gramd1b*, *Stard3*, and *Npc1/2*) and storage (*Soat 1/2*). Moreover, we found that IFNs induced the accumulation of a small amount of cholesterol esters in macrophages. Inhibiting ACAT enzymes increased the sensitivity of macrophages to CDCs, even in the absence of CH25H. Thus, we proposed that IFNs induce a robust but highly selective cholesterol redistribution program that moves cholesterol targeted by CDCs out of the PM, redistributes it to the ER, and subsequently stores esterified cholesterol if needed. Inhibiting cholesterol synthesis with 25HC is necessary to prevent this small but highly labile pool from refilling and resensitizing macrophages to CDC toxins. A working model of how IFN-mediated reprogramming of cholesterol homeostasis promotes resistance to CDCs is shown in Fig. 4, however many of the mechanistic details of this proposed model need to be better defined and tested.

 A recent complementary study showed that IFN-mediated production of 25HC increased cellular immunity to *L. monocytogenes* and *Shigella flexneri* by inhibiting cell–cell spreading [34]. This study found that 25HC interfered with the ability of these microbes to traverse the plasma membrane of infected cells and enter uninfected neighboring cells through double plasma membrane structures. The molecular mechanism is also under investigation but appears to be dependent on rapid internalization of the accessible cholesterol pool via the activation of ACATs and subsequent storage of this cholesterol as esters in a process that is highly analogous to that seen in macrophages [34]. However, it is important to note that 25HC was not effective at blocking *L. monocytogenes* escape from the phagocytic vacuole, which is a process that requires listeriolysin O (LLO), a CDC produced by these microbes. Therefore, the mechanism by which IFN-induced changes in the accessible cholesterol pool alter CDC sensitivity may only be relevant to the plasma membrane., Nevertheless, this study reinforces the growing concept that reprogramming the pool of accessible plasma membrane cholesterol with IFNs can protect cells from damage induced by microbes.

Based on these new concepts in membrane cholesterol homeostasis, it will be interesting to revisit whether other intracellular microbes that exploit cellular cholesterol for their lifecycle depend on the accessible cholesterol pool. Given that both type I and type II IFNs regulate the size of the metabolically active cholesterol pool, we suspect that this small pool of cholesterol will also be necessary for viruses that rely on cholesterol for entry. 25HC has been shown to block viral entry [22, 23, 63], so it is possible that 25HC-mediated regulation of  the metabolically active cholesterol pool in the PM is one of the molecular mechanisms underlying the antiviral effect of 25HC.

## Future directions: necrotizing fasciitis and a speculative link to cholesterol metabolism

Necrotizing fasciitis (NF), also known as flesh-eating disease, is a subset of an aggressive skin and soft tissue infection consisting of liquefying necrosis of dermal and subcutaneous tissues [64]. NF is mediated by select gram-positive microbes that secrete toxins, including CDCs and hemolytic toxins into infected and surrounding tissues [65]. The observation that metabolic reprogramming of cholesterol metabolism attenuates CDC-mediated cytotoxicity and tissue damage in the skin is striking. It is tantalizing to hypothesize that the dysregulation of tissue lipid homeostasis could influence the extent of tissue damage associated with necrotizing soft tissue infection., Development of NF is rare and it remains unclear why individuals develop NF. However, it is worth noting that comorbidities associated with the development of NF include metabolic diseases, such as obesity and diabetes [66, 67]. Thus, it is possible that the dysregulation of lipid metabolism, in particular cholesterol homeostasis, sensitizes individuals to the deleterious effects of CDCs and other toxins that drive the pathogenesis of NF. It will be necessary for the field to test these  interesting but nascent ideas. Likewise, it will be exciting to determine whether targeting lipid metabolism in infected tissues attenuates the development of NF, particularly in individuals who have pre-existing lipid metabolic dysregulation. If proven true, this concept will open new avenues for developing adjunctive therapies to attenuate these rare but highly pathogenic skin and soft tissue infections.

## Conclusions

It is now clear that reshaping lipid composition is an integral and essential part of myeloid cell differentiation and function. In the absence of proper lipid metabolic reprogramming, macrophages exhibit dysregulated inflammatory responses and altered immune functions. These observations suggest that environmental or metabolic signals that interfere with the metabolic reprogramming of lipid composition will result in phagocyte dysfunction. An additional layer of complexity lies in the observation that macrophages do not converge on a single lipidome irrespective of the activating signal. Instead, distinct proinflammatory stimuli drive the acquisition of different lipidomes [17]. These data indicate considerable specificity in  the lipidome that macrophages acquire during different inflammatory responses. Moreover, these specific changes in lipid composition appear to impart information to the cell that ultimately regulates  distinct effector functions and immunity. We expect that there will be instances in which reprogramming of lipid composition will be beneficial for some forms of immunity but harmful to other forms of immunity. For example, reprogramming of cholesterol metabolism may benefit antiviral immune responses but interfere with antimicrobial responses. This additional layer of complexity also suggests that a context-specific approach to correcting the metabolism of macrophages and other immune cells will be necessary if one hopes to normalize function.

Of course, there remain many important and unresolved questions about lipid metabolic reprogramming that the field of immunometabolism should address. One crucial issue is to determine the extent to which activation signals reshape lipid composition in the context of infections. Much of our knowledge about lipid metabolic reprogramming is predicated on knowledge gained using highly reductionist systems. The intrinsic complexity of infections will undoubtedly muddy the water of our current working models. We predict that there will be instances in which pathogens misdirect macrophages to acquire the wrong lipidome, ostensibly interfering with requisite effector functions to clear infections. It will be exciting for the field to generate comprehensive pathogen-based immune metabolic studies to guide our thinking and shape models.

Another series of questions for the field to address center around the issues of durability and plasticity. The approaches we and others have taken reasonably focus on short-lived inflammatory macrophages. It remains unclear how durable these changes in lipid composition are and whether macrophages can undergo secondary reshaping of their lipidome in response to newly received information. For example, we envision that exposure to different cytokines throughout an immune response will continually reshape the lipidome to match required effector functions.  . Alternatively, it is possible that initial exposure to a specific proinflammatory cytokine (i.e., IFN-γ) may render cells refractory to any changes in lipid composition, essentially removing any plasticity in metabolism. Macrophages have variable lifespans, and resident tissue macrophages are long-lived with some self-renewal capabilities [3]. It will be important to determine whether inflammation-driven metabolic reprogramming of lipid metabolism indelibly imprints on long-lived macrophages, their progeny in tissues, or myeloid stem cells. If such an observation was found to be true,  this could be critical for understanding pathogenic circuits that link metabolic disease (e.g., atherosclerosis) and inflammation in apparent feed-forward systems. This type of indelible programming could also help to explain aspects of innate immune memory or the capacity of innate immune cells to generate preferential immunity to pathogens upon subsequent exposures. Tackling these exciting and important questions will undoubtedly advance our mechanistic understanding of immunometabolism. We also believe that continued research into the crosstalk between lipid metabolism and the function of macrophages will provide essential insights for developing new therapeutic approaches to control unwanted inflammation, infections, and metabolic diseases.
